# Plastic and low-cost axial zero thermal expansion alloy by a natural dual-phase composite

**DOI:** 10.1038/s41467-021-25036-1

**Published:** 2021-08-04

**Authors:** Chengyi Yu, Kun Lin, Suihe Jiang, Yili Cao, Wenjie Li, Yilin Wang, Yan Chen, Ke An, Li You, Kenichi Kato, Qiang Li, Jun Chen, Jinxia Deng, Xianran Xing

**Affiliations:** 1 Beijing Advanced Innovation Center for Materials Genome Engineering, Institute of Solid State Chemistry, Department of Physical Chemistry, University of Science and Technology Beijing, Beijing, China; 2 Neutron Scattering Division, Oak Ridge National Laboratory, Oak Ridge, TN USA; 3 RIKEN SPring-8 Center, Hyogo, Japan

**Keywords:** Chemistry, Materials science

## Abstract

Zero thermal expansion (ZTE) alloys possess unique dimensional stability, high thermal and electrical conductivities. Their practical application under heat and stress is however limited by their inherent brittleness because ZTE and plasticity are generally exclusive in a single-phase material. Besides, the performance of ZTE alloys is highly sensitive to change of compositions, so conventional synthesis methods such as alloying or the design of multiphase to improve its thermal and mechanical properties are usually inapplicable. In this study, by adopting a one-step eutectic reaction method, we overcome this challenge. A natural dual-phase composite with ZTE and plasticity was synthesized by melting 4 atom% holmium with pure iron. The dual-phase alloy shows moderate plasticity and strength, axial zero thermal expansion, and stable thermal cycling performance as well as low cost. By using synchrotron X-ray diffraction, in-situ neutron diffraction and microscopy, the critical mechanism of dual-phase synergy on both thermal expansion regulation and mechanical property enhancement is revealed. These results demonstrate that eutectic reaction is likely to be a universal and effective method for the design of high-performance intermetallic-compound-based ZTE alloys.

## Abstract (web summary)

Zero thermal expansion alloys have unique dimensional stability but suffer from inherent brittleness and compositional sensitivity. Here the authors present the one-step eutectic reaction approach to produce a dual-phase alloy with an axial zero expansion and promising mechanical properties.

## Introduction

Zero thermal expansion (ZTE) alloy plays an important role in our daily life ranging from mechanical watches to communication satellites because of its size-stability under temperature flucuations^1–3^. However, ZTE material is rare in nature. This behavior is especially unusual for metallic materials. Only a few couplings among lattice, spin, and orbital induce a reduced coefficient of thermal expansion by the virtue of the so-called magneto-volume effect^4–6^, which has been reported in a few single-phase metallic materials, such as the conventional Invar alloy^7^ (Fe_0.64_Ni_0.36_) and some magnetic intermetallic compounds (e.g., Tb(Co,Fe)_2_^8^, La(Fe,Si,Co)_13_^9^). On the other hand, most of these ZTE compounds are brittle with low strength and little ductility^10–14^, with concerned applicability due to low fracture toughness. It is noted here that the widely used Invar alloy shows plasticity but with low strength. An alternative way to design ZTE alloys is to mix thermal expansion material with thermal contraction one (e.g., La(Fe,Si)_13_/Cu^15^ and ZrW_2_O_8/_Al^16^). But these artificial composites usually suffer from undesired microstructures or weak interfacial bonding, resulting in poor overall mechanical properties and thermal cycling performances. More importantly, the magneto-volume effect is highly composition-sensitive—a slight interfacial mass transfer during high-temperature synthesis may suppress or vanish the ZTE property^5,13,17^. The search for novel ZTE alloys with excellent strength-plasticity performance has been challenging for decades^18–22^.

In this work, we conducted a one-step strategy to design plastic and low-cost ZTE alloy by a hypo-eutectic or hyper-eutectic reaction in a binary system^23–25^. Iron is one of the most earth-abundant elements; its conventional phase, α-Fe, has high plasticity and normal positive thermal expansion (PTE). Interestingly, on the R-Fe (R = rare earth) binary phase diagram^26^ (see Supplementary Fig. 1), Fe forms a eutectic system with R_2_Fe_17_—a typical negative thermal expansion (NTE) intermetallic compound driven by magnetic ordering^6,27,28^. This indicates that the R_2_Fe_17_ phase could coexist in equilibrium with Fe at any temperature without losing its own NTE character. What’s more, both the phase fraction and microstructure can be easily controlled by tuning the chemical compositions in the binary system, which are key factors to thermal expansion and mechanical property improvement. Here we demonstrate that by adding only 4% Ho atoms to pure iron, an alloy with both axial ZTE (Ho_0.04_Fe_0.96_, *α*_l _= 0.19 × 10^−6^ K^−1^, 100 to 335 K) could be designed and fabricated and a moderate strength-plasticity combination could be achieved. We further show that the present dual-phase alloy is highly stable under thermal circulation conditions, which is rare among alloy materials that possessed both ZTE and plasticity, and the cost-effective alloy sees great potential in applications.

## Results

### Phase and crystal structure

**Fig. 1:**
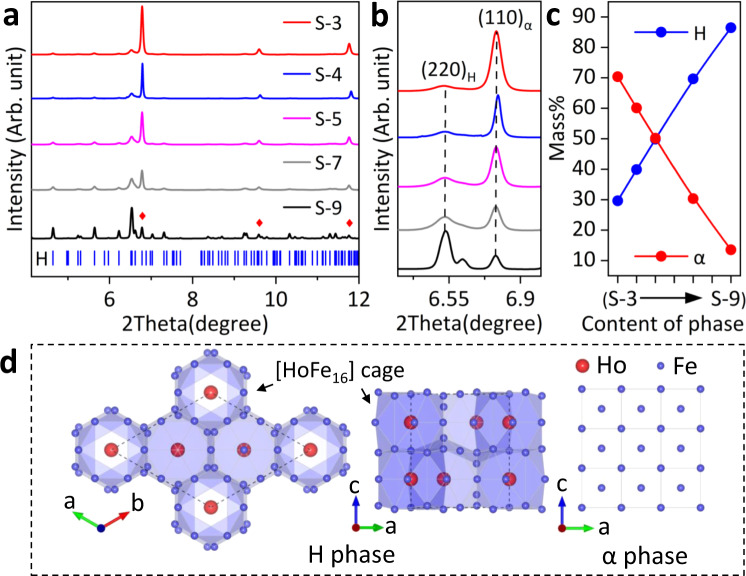
Phase and crystal structures. **a**, **b** High-resolution synchrotron X-ray diffraction profiles for S-3 to S-9 (*λ* = 0.23991 Å), the marked red diamond is the α phase. **c** The mass fractions of H and α in S-3 to S-9 are determined via Rietveld refinements. **d** Crystal structures of H and α phase, respectively.

The targeted samples with compositions of Ho_*x*_Fe_1*−x*_ (*x* = 0.03, *x* = 0.04, *x* = 0.05, *x* = 0.07, *x* = 0.09, labeled as S-3 to S-9) were synthesized by traditional arc-melting. High-resolution synchrotron X-ray powder diffraction (SXRD) measurement was employed to identify the phase fractions and crystal structures. It shows that all the samples are dual-phase alloys consist of the Ho_2_Fe_17_ phase (denoted as H) and α iron phase (denoted as α) without other detectable impurities (see Fig. 1a). Increasing the content of Ho, the positions of Bragg peaks for both the H and α phases keep almost unchanged as indicated by (220)_H_ and (110)_α_ reflections in Fig. 1b, suggesting that the two phases reached thermodynamic equilibrium and can coexist in any proportion to ensure desired thermal expansion regulation. These results agree well with the Ho–Fe binary phase diagram.

Figure 1d shows the crystal structure of the H and α phases refined by SXRD data. The H phase adopts Th_2_Ni_17_-type structure (space group: *P*6_3_/*mmc*) with cell parameters *a* = 8.45 ± 0.01 Å, *c* = 8.32 ± 0.01 Å, *V* = 514.09 ± 0.03 Å^3^ and has six Wyckoff sites (Ho_2b_, Ho_2d_, Fe_4f_, Fe_6g_, Fe_12j_, and Fe_12k_); while the α phase is a body-centered-cubic phase (space group: *I m* − 3*m*) with cell parameters *a* = 2.87 ± 0.00 Å, *V* = 23.56 ± 0.02 Å^3^ and has only one Wyckoff site (Fe_2a_). Due to a large difference in atomic radius, Ho (1.79 Å) and Fe (1.26 Å) atoms occupy separate sites in the H phase selectively: Ho atoms are surrounded by Fe atoms and form [HoFe_16_] cages (Fig. 1d). This guarantees the structural stability of the H phase when Fe atoms can diffuse through the interface during the high-temperature synthesis. Further Rietveld refinements quantified the phase fractions of the H phases (29.6 ± 0.1%, 39.8 ± 0.1%, 49.8 ± 0.1%, 69.7 ± 0.2%, and 86.5 ± 0.3% for S-3 to S-9, respectively, see Fig. 1c and Supplementary Fig. 2).

### Microstructure and crystallographic orientation

**Fig. 2:**
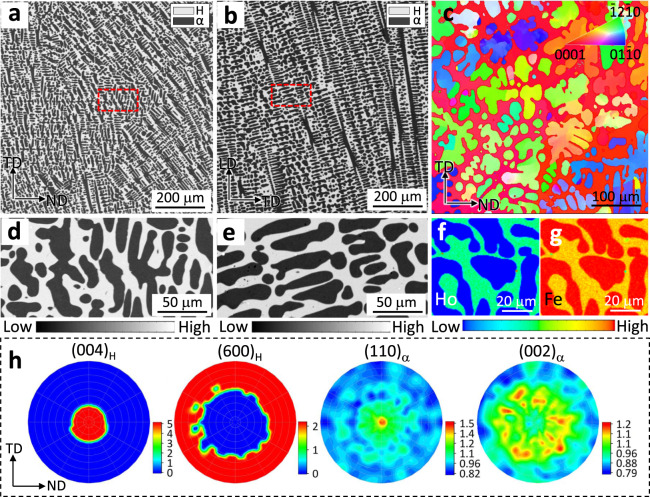
Microstructures of the ZTE alloy. **a**–**b**, **d**–**e** The morphology of the as-cast S-4 alloy confirmed by electro-probe microanalyzer (EPMA) in TD (transverse direction)-ND (normal direction) plane (**a**) and LD (loading direction)-TD plane (**b**), respectively. (**d**) and (**e**) are enlarged regions in (**a**) and (**b**) marked with a red box. **c** Electron back-scattered diffraction (EBSD) inverse pole figure of crystal orientation for S-4 inside the TD-ND plane. **f**–**g** Element mappings of Ho (**f**) and Fe (**g**). **h** Pole figures by neutron diffraction texture analysis for the bulk orientations of (004)_H_, (600)_H_, (110)_α_, and (002)_α_ directions.

Electro-probe microanalyzer (EPMA) shows that the dendritic lamellar α phase (in dark, 50–100 μm) is homogeneously dispersed into the H phase (in white, 50–100 μm) matrix (see Figs. 2a–2e for S-4, see Supplementary Fig. 3 for other samples). Besides, the grains appear to grow along the loading direction (LD) (see Fig. 2b). Electron back-scattered diffraction (EBSD) inverse pole image demonstrates that the H and α phases are highly textured (Fig. 2c). This is caused by the large temperature gradient and cooling rate in the cooling process, in which the phases tend to nucleate and grow along a certain direction and form bulk materials with strong anisotropy. To further investigate the lattice matching between the two phases in the bulk, neutron diffraction texture analysis was carried out. The pole figures demonstrate a strong fiber texture in the as-cast sample: the H phase is highly textured with the {004} grains are parallel to the loading direction (LD) while the {600} grains are perpendicular to LD; the α phase has a less-strong orientation with [110]_α_ roughly parallel to LD (see Fig. 2h). Consequently, the main orientation relationship between the H and α phase is [001]_H_//[110]_α_. This facilitates the formation of semi-coherent matching at the dual-phase interface and will be discussed later.

### Thermal expansion and mechanical properties

**Fig. 3:**
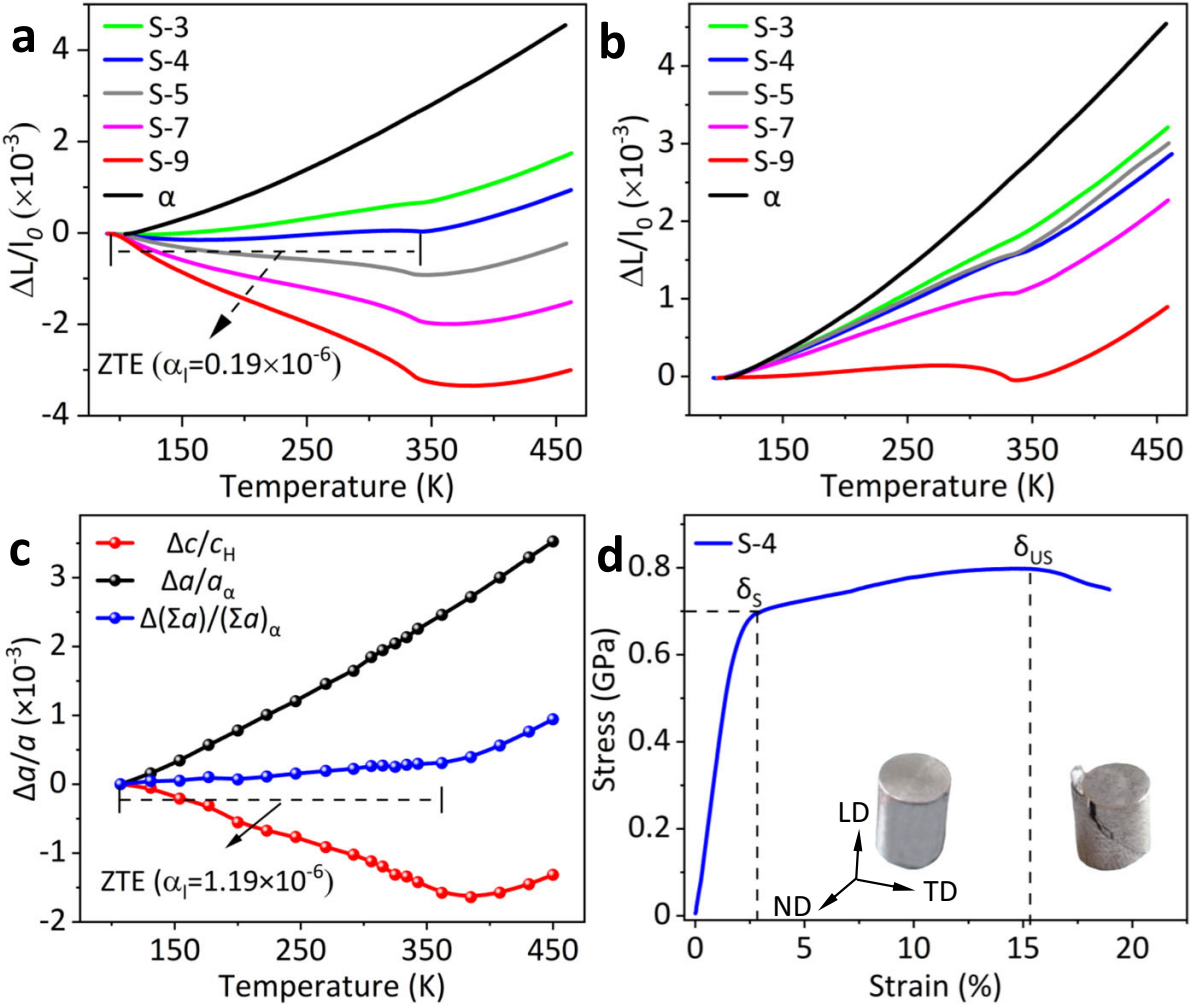
Thermal expansion and mechanical properties. **a** Linear thermal expansion determined by advanced thermo-dilatometer for S-3 to S-9 and iron along with LD. **b** The in-plane (TD-ND) linear thermal expansion of S-3 to S-9 and iron. **c** Lattice thermal expansions of α along the *a* axis, H along the *c* axis, and S-4 along with LD. **d** Compressive stress-strain curves of the S-4 with the insets of S-4 ingot during loading.

The hexagonal H compound shows NTE but is brittle, while the bcc α phase shows positive thermal expansion (PTE) and is plastic. Hence, both the coefficient of thermal expansion (CTE) and mechanical properties of the dual-phase alloys could be well controlled by adjusting phase content. Figure 3a indicates that the linear thermal expansion along LD of alloys can be successively tailored from moderate positive (*α*_l _= 2.7 × 10^−6^ K^−1^, S-3) to strong negative (*α*_l _= −12.9 × 10^−6^ K^−1^, S-9). Especially, an axial ZTE over a wide temperature range (100 to 335 K) has been obtained in S-4 alloy (*α*_l _= 0.19 × 10^−6^ K^−1^). Such a ZTE performance demonstrates dimensional stability even under hundreds of thermal cycling conditions (see Supplementary Fig. 4 and 5). Due to the strong texture in the alloys, the CTEs in-plane (TD-ND) from S-3 to S-9 are varied from 7.7 × 10^−6^ K^−1^ to −0.1 × 10^−6^ K^−1^ (see Fig. 3b). The lattice thermal expansions in the S-4 alloy were extracted by the temperature resolved SXRD, which are 9.40 × 10^−6^ K^−1^ for α phase along the *a* axis and −5.91 × 10^−6^ K^−1^ for H phase along the *c* axis (see Supplementary Fig. 6 and Table 2). This leads to an overall thermal expansion along the LD of the alloy to be 1.19 × 10^−6^ K^−1^ in the temperature range of 100 to 335 K (Σ*a*_S-4_ = mol._α_% × *a*_α_ + mol._H_% × *c*_H_, see Supplementary Methods), consistent with that of dilatometer measurement (see Fig. 3c). What’s more, S-4 displays good plasticity during compressive loading, as shown in the engineering stress-strain curve in Fig. 3d. The ultimate compressive stress (*δ*_US_) is up to 0.80 ± 0.02 GPa and the alloy undergoes almost 15.5% compressive strain with an obvious strain-hardening before failure. We note that although S-9 shows ZTE performance in the ND-TD plane, this alloy is brittle with low strength (*δ*_US_ = 0.41 ± 0.01 GPa, see Supplementary Fig. 7), similar to many other ZTE intermetallic compounds^5–11^. Thereafter, the S-4 alloy will be comprehensively discussed because of its ZTE behavior and plasticity. To date, such a high strength-plasticity combination has been scarcely achieved among known ZTE or low thermal expansion (LTE) metallic materials^5–12,15,18^.

## Discussion

**Fig. 4:**
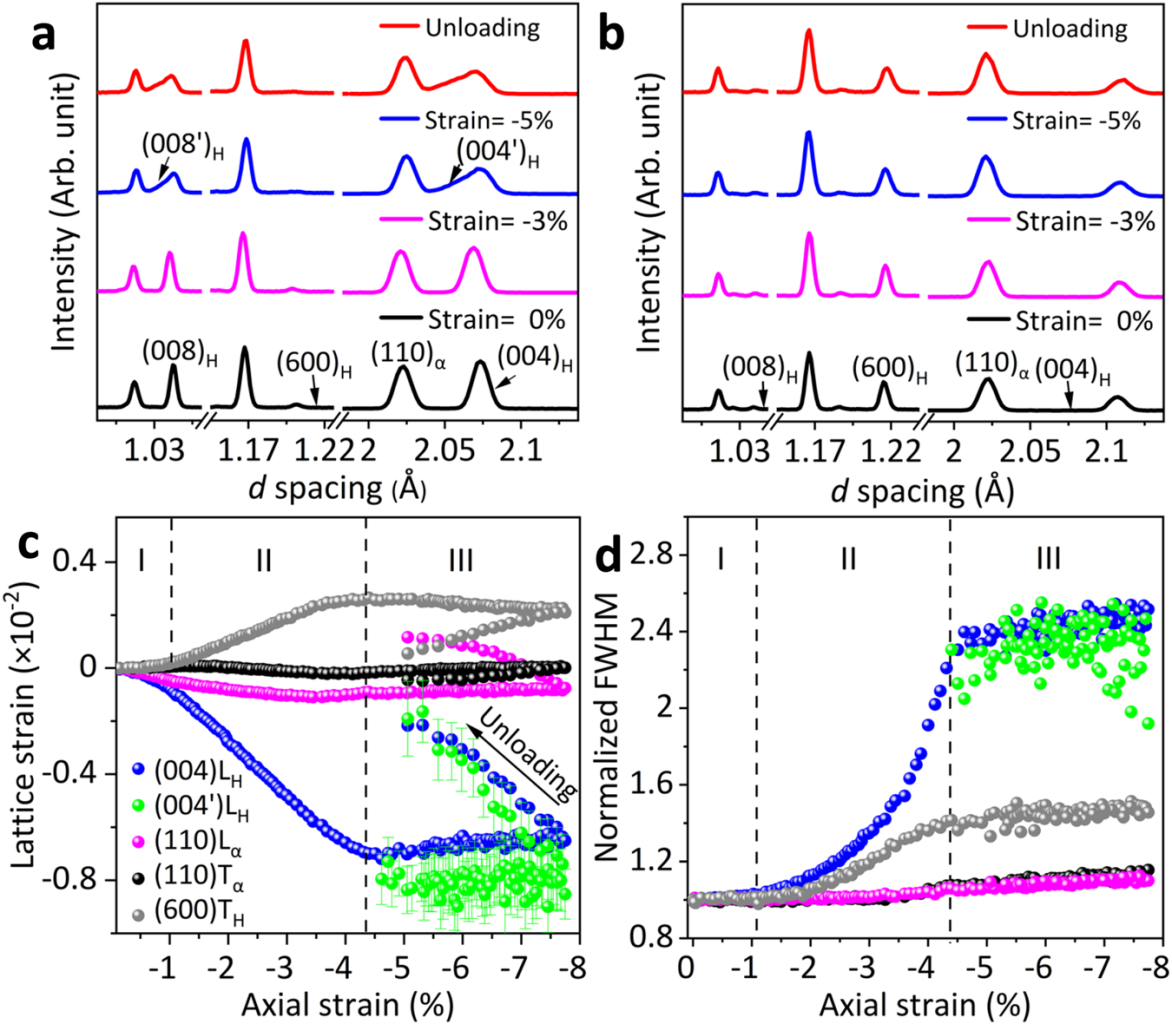
Real-time in-situ neutron diffraction studies of S-4 alloy. **a**, **b** In-situ neutron diffraction profiles at the strain of 0%, −3%, −5%, and unloading stage collected in the LD (**a**) and TD (**b**), respectively, correspond to I, II, and III stages. **c**, **d** Lattice strains (**c**) and normalized peak FWHMs (**d**) in LD and TD versus applied compressive strain, respectively.

To shed light on the mechanism for enhanced strength and plasticity in the ZTE alloy, we investigated the co-deformation process of the two phases by in-situ neutron diffraction measurements under uniaxial compression^29,30^ (see Supplementary Fig. 8). Two sets of neutron diffraction patterns were simultaneously recorded in the longitudinal direction (LD, along loading direction) and transverse direction (TD, perpendicular to loading direction) upon loading (see Supplementary Fig. 9). The dramatic difference in peak intensities between LD and TD directions for both the H and the α phases is caused by fiber texture in the samples, in agreement with previous microstructure analysis (Figs. 4a and 4b). The lattice strain evolution with applied axial strain includes three stages (see Fig. 4c): the soft α and brittle H phases co-deformed elastically in stage I; the α phase deformed plastically and the H phase still maintained elastically in stage II, the α phase yielded at about 0.15 GPa, which is consistent with the behavior of pure α-Fe, indicating that the H phase has little effect on its yielding behavior (see Supplementary Fig. 10); eventually, co-deformed plastically in stage III.

Figure 4c demonstrates that the interplanar space of (004)_H_ can be continuously compressed by increasing engineering strain up to −4.4%, corresponding to the elastic deformation of the H phase at stage II along with a gradually yielding of the α phase. We extracted the interaction between the soft α and the brittle H phases by illustrating the full width at half maximum (FWHM) of the reflections (Fig. 4d): the slightly broadening of (110)_L_ and (110)_T_ in the α phase corresponds to dislocations generation during yielding (L and T represent LD and TD, respectively); however, the prominently broadening of (004)_L_ in the H phase during elastic deformation corresponds to the accumulation of large local strain gradient along with the nucleation of shear bands. It should be emphasized that although both phases yielded and plastically deformed in stage III, the Th_2_Ni_17_-type H phase with large unit cell (*V* = 514.09 ± 0.03 Å^3^) and low symmetry (*P*6_3_/*mmc*) lacks enough independent slip systems for plastic deformation. In fact, the plasticity of the H phase in Stage III is driven by the shear band mechanism^21,31–35^ (see Supplementary Fig. 11). This is verified by the asymmetric appearance of the {001}_H_ reflections along with LD in this stage (arrows in Fig. 4a and Supplementary Fig. 12)—there exist widespread lattice strains caused by shear bands. That is, the lamellar morphology from the eutectic reaction enhances the alloys’ mechanical performance via dual-phase synergetic interaction^20,23,31,36–39^.

**Fig. 5:**
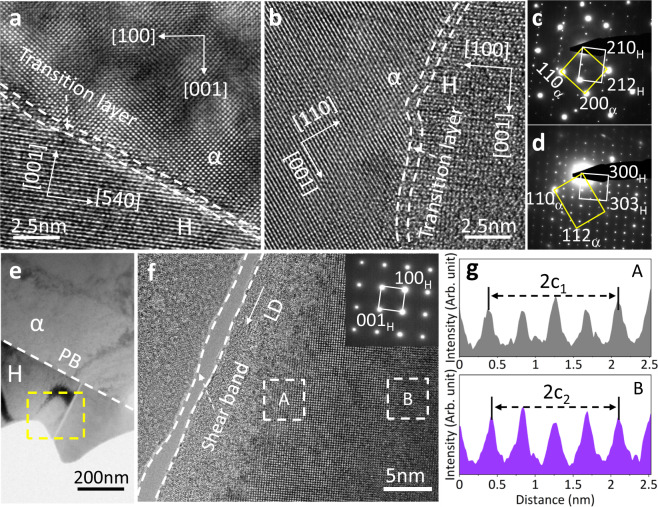
TEM studies of S-4 alloy. **a**, **b** High-resolution transmission electron microscopy (HRTEM) image at the phase interface, oriented to the [001]_α_ zone axis (**a**) and [1$\overline{1}$0]_α_ zone axis (**b**), respectively. **c**, **d** Selected area electron diffraction (SAED) at the phase interface correspond to Fig. 5a and b, respectively. **e** The microstructure of the S-4 alloy at the strain of −5% along the loading direction, the white dashed line indicates the phase boundary (PB). **f** The HRTEM image at the shear band area of the H phase, insert is the SAED at the H phase, oriented to the [010] zone axis. The local shear band is formed after large compressive deformation. **g** Intensity profile along with LD in A and B zones marked in Fig. 5f.

Phase interface also plays an important role in the enhanced mechanical behaviors and thermal cycling stability^40–42^. High-resolution TEM reveals a ~1 nm thick disordered transition layer at the interfaces as shown in Figs. 5a and 5b, two typical interfacial morphologies. The orientation relationship at these interfaces are ∠([110]_α_, [001]_H_) = 34.7° and [112]_α_//[001]_H_ (Figs. 5c and 5d), respectively, implying that there exist some extent of semi-coherent lattice matching between the two phases. The transition layers connect H and α phases via chemical bonding, minimalizing interfacial energy and giving rise to strong interface linkage and stable thermal cycling performance^43,44^. The thermal cycling test shows that the dual-phase ZTE alloys remain in perfect integrity after hundreds of rapid switching between 77 K and 335 K (see Supplementary Fig. 13). Besides, the high elastic strain energy of the brittle H phase can be effectively transferred to the soft α phase upon loading, and be relieved via dislocation multiplication in the soft and plastic α phase. This improves the alloy’s thermal cycling performance and mechanical stability. Figure 5f demonstrates a typical shear band in the H phase (along loading direction) after −5% deformation, where the lattice constant *c* near the shear band at region A (8.46 Å) is smaller than that away from it at region B (8.57 Å). The heterogeneous lattice strain in the H phase (see Figs. 5e–3g) caused by interface interaction accounts for the asymmetric change of the {001}_H_ reflections obtained by in situ neutron diffraction (Fig. 4a).

This dual-phase synergistic interaction mediated by interface was further demonstrated by ex-situ microstructure studies. After −12% deformation, the localized shear micro-cracks in the brittle H phase are inhibited by the soft α phase due to its lamellar structure (see Supplementary Fig. 11). Hence, the α dendrites act as obstacles hindering the excessive deformation of the H phase by pinning the highly localized shear micro-cracks. Meanwhile, substantial dislocations generate in the plastic α phase after deformation to −12% strain (see Supplementary Fig. 14), which eliminates the stress concentration of the hard H phase and makes it plastic and robust. With further strain increase, the micro-cracks divide into sub-micro-cracks along phase boundaries and lead to shear failure by the adjacent micro-crack penetration (see Supplementary Fig. 15). These results illustrate that the dual-phase alloy undergoes large deformation by absorbing a large amount of work from fracturing, which improves the overall toughness and brings plasticity to the dual-phase alloy.

**Fig. 6:**
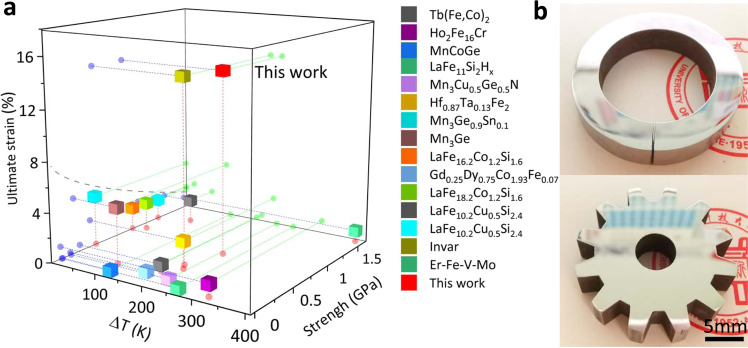
Summary of mechanical and thermal expansion performance. **a** A review of critical parameters for the typical (near) zero thermal expansion metallic materials: ultimate strain, strength, and temperature window. Note: Invar is a completely plastic material, for comparison, we used the compressive strength at 15.6% strains here. **b** Pictures of gear (up) and sealing (down) ring fabricated by the present ZTE alloy (S-4).

For comparison, in Fig. 6a we summarized ultimate strain versus compressive strength and ZTE temperature range (ΔT) for the ZTE alloy S-4, and other typical ZTE or near ZTE metallic materials^5,7,8,11,12,15,18,45–49^. ZTE intermetallic compounds are of low strength and high brittleness though they possess desired ZTE performance. For traditional La(Fe,Si)_13_-based composites, their strengths are high but have little plasticity and their ZTE is mostly limited in a narrow temperature range. Er-Fe-V-Mo dual-phase alloy has high strength and wide near ZTE temperature range, but has no plasticity. In contrast, the present dual-phase ZTE alloy S-4 shows a favorable strength-plasticity combination and a considerable wide ZTE temperature range (Δ*T* = 235 K) and is much stronger than the widely used Invar alloy at comparable strains. Most importantly, this ZTE alloy S-4 is easy to machine and can be fabricated to various structures, such as precision gears against thermal shock and sealing ring enduring temperature fluctuation, etc., with stable thermal cycling performance (see Fig. 6b and Supplementary Fig. 5). We also emphasize that the present Fe-based ZTE alloy contains only 4 at.% rare-earth Ho, making it cost-effective with great application potentials (see Supplementary Fig. 16).

In summary, we used the eutectic reaction method to overcome the contradiction between ZTE and plasticity in metallic materials. By this method, we successfully designed and fabricated a Fe-based ZTE dual-phase alloy, Ho_0.04_Fe_0.96_, with a combination of low-cost, stable thermal cycling performance, and moderate strength-plasticity. The lamellar dual-phase microstructure with the semi-coherent interface not only regulates the thermal expansion behavior but also greatly enhances the mechanical properties and improves the thermal stability of the Fe-based ZTE alloy. The comprehensive performance of the present dual-phase alloy may effectively avoid the “Buckets Effect” caused by the imbalance of material properties, endowing it a broad application prospect. We expect that more high-performance ZTE alloys could be developed by using the eutectic reaction strategy.

## Methods

### Synthesis methods

The samples of Ho_*x*_Fe_1−*x*_ (*x* = 0.03, *x* = 0.04, *x* = 0.05, *x* = 0.07, *x* = 0.09, labeled as S-3, S-4, S-5, S-7, S-9) were prepared the constituent elements, Ho and Fe (>99.9% purity) by a vacuum arc melting furnace under high purity argon atmosphere. The samples were turned over and melted four times to ensure homogeneity. Then, the sample was followed by annealing at 1373 K in an argon atmosphere for about 24 h and quenched in water.

### SXRD measurements

The ambient temperature (*λ*= 0.23991 Å) and in-situ SXRD measurements (*λ*= 0.45 Å) of the samples were performed at the SPring-8, Japan. The phase structures and fractions were obtained by Rietveld refinements with FULLPROF software.

### EPMA measurements

The phase contrast and microstructure analysis were measured by electro-probe microanalyzer backscattering electron (EPMA-BSE) spectrum (SHIMADZU 1720) equipped with wave-length dispersive spectrometer analysis (WDS) to quantitative determine the phase composition.

### SEM and EBSD measurements

The surface of fracture and the microstructure orientation of the samples were measured by scanning electron microscope (SEM, Zeiss Geminisem 500), electron backscattering diffraction (EBSD, TESCAN MIRA 3 LMH SEM, and Symmetry EBSD).

### TEM measurements

The microstructure of the as-cast sample was characterized by the high-resolution transmission electron microscopy (HRTEM) and was measured by an aberration-corrected FEI Titan G260–300 kV S/TEM. All pictures were handled with Gatan Digital Micrograph software.

### Linear thermal expansion of the alloy

The linear thermal expansion curves (Δ*L*/*l*_0_) were measured by an advanced thermo-dilatometer (NETZSCH DIL402) with a heating rate of 5 K/min.

### Mechanical properties

The sample was fabricated into Φ 6 × 8 mm cylinder by electrical discharging. At least 5 samples were tested for each composition. The room-temperature mechanical properties were measured using a CMT4105 universal electronic compressive testing machine with an initial strain rate of 7.0 × 10^−4^ s^−1^.

### In situ neutron diffraction measurements

The real-time in-situ neutron diffraction experiments were carried out at VULCAN beamline in Oak Ridge National Laboratory^29,50^ (ORNL), USA. All the lattice strain during the compressive is determined by the single peak fitting method for the (h,k,l) reflections. The lattice strain was calculated by following formula (Eq. 1):

1 $${{{{{\rm{Strain}}}}}}=({d}_{1}-{d}_{0})/{d}_{0}\times 100 \%$$

Here *d*_1_ and *d*_0_ represent the interplanar crystal spacing of the (hkl) crystal plane after and before loading, respectively.

### The lattice thermal expansion of the dual-phase alloy

The lattice thermal expansion of dual-phase alloy was calculated as (Eq. 2) and (Eq. 3):

2 $${\alpha }_{S-4}=\frac{\Sigma {\alpha }_{1}-\Sigma {\alpha }_{0}}{\Sigma {\alpha }_{0}}/({T}_{1}-{T}_{0})$$

3 $$\Sigma {a}={{{{{\rm{mol}}}}}}{.}_{{{{\it{\alpha }}}}} \% \times {{a}}_{{{{\it{\alpha }}}}}+{{{{{\rm{mol}}}}}}{.}_{{{{{{\rm{H}}}}}}} \% \times {{{{{{\rm{c}}}}}}}_{{{{{{\rm{H}}}}}}}$$

where α_S-4_ is the corrected CTEs along with the LD for S-4; mol._α_% and mol._H_% are molar fractions of α and H determined by the results of SXRD data. Due to the high crystallographic texture of the S-4 with <001>//LD and the homogeneous lamellar microstructure, we use the *c* axis of the H phase, *a* axis for the α phase, and molar fractions to calculate the overall thermal expansion along with the LD.

### Thermal cycling test

To determine the thermal cycling stability of the dual-phase alloys caused by the mismatch of CTEs between the two phases, a thermal-cycling test was conducted by an auto-mechanical arm. The sample was submerged in liquid nitrogen (77 K) for 10 s and then quickly transferred to hot water (335 K) and kept for another 10s, which is the whole cycle.

## Supplementary information

Supplementary Information

## Data Availability

The data that support the findings of this study are available from the corresponding authors upon reasonable request.
