# Supplementary material for: Plastic and low-cost axial zero thermal expansion alloy by a natural dual-phase composite
Source: Nat Commun. 2021 Aug 4;12:4701. doi: 10.1038/s41467-021-25036-1 (PMC8338949; doi:10.1038/s41467-021-25036-1)
Supplement: Supplementary file 1 — Supplementary Information [file 41467_2021_25036_MOESM1_ESM.pdf]

Supplementary Information

Plastic and low-cost axial zero thermal expansion alloy by a natural dual-phase composite

Chengyi Yu,[1] Kun Lin,[1,*] Suihe Jiang,[1] Yili Cao,[1] Wenjie Li,[1] Yilin Wang,[1] Yan Chen,[2] Ke An,[2] Li You,[1] Kenichi Kato,[3] Qiang Li,[1] Jun Chen,[1] Jinxia Deng,[1] Xianran Xing,[1,*]

1. Beijing Advanced Innovation Center for Materials Genome Engineering, Institute of Solid State Chemistry, Department of Physical Chemistry, University of Science and Technology Beijing, Beijing 100083, China

2. Neutron Scattering Division, Oak Ridge National Laboratory, Oak Ridge, TN 37831, USA.

3. RIKEN SPring-8 Center, Hyogo 679-5148, Japan.

**Corresponding author: xing@ustb.edu.cn; kunlin@ustb.edu.cn*

This PDF file includes:

1. Supplementary Figure 1-16.
2. Supplementary Table 1-2.

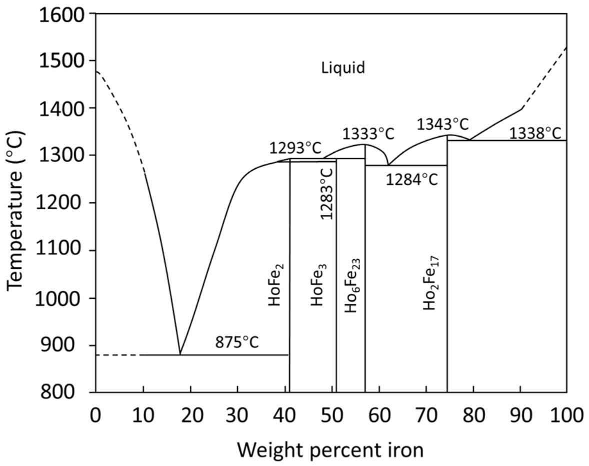

Supplementary Fig. 1. The Fe-Ho phase diagram.

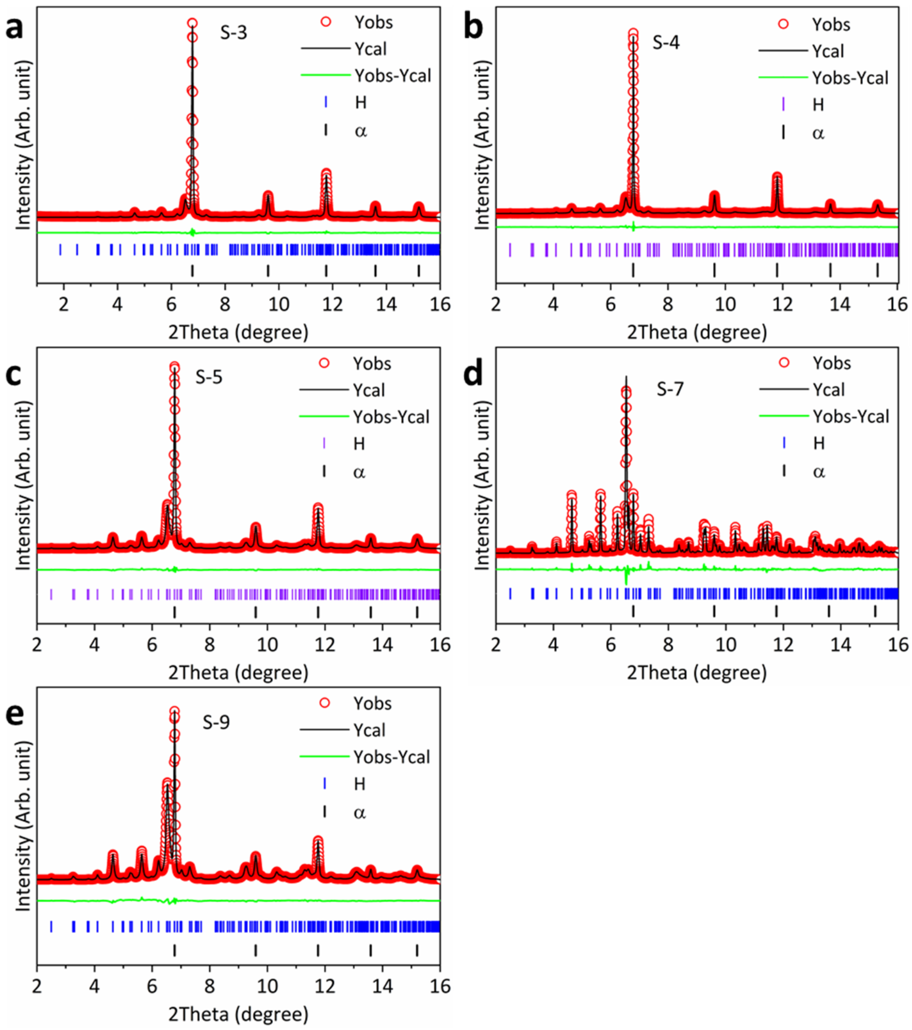

Supplementary Fig. 2. The Rietveld refinements patterns for S-3 (**a**), S-4 (**b**), S-5 (**c**), S-7 (**d**) and S-9 (**e**), respectively.

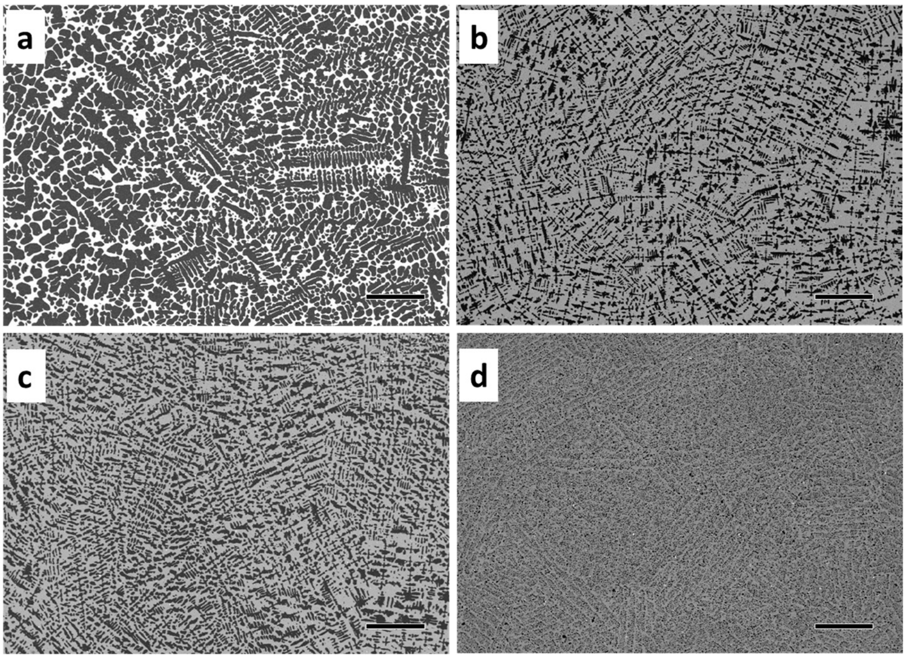

Supplementary Fig. 3. The morphology of the as-cast alloy S-3 (**a**), S-5 (**b**), S-7 (**c**) and S-9 (**d**) by EPMA in the TD -ND plane. All scale bars represent 150 μm.

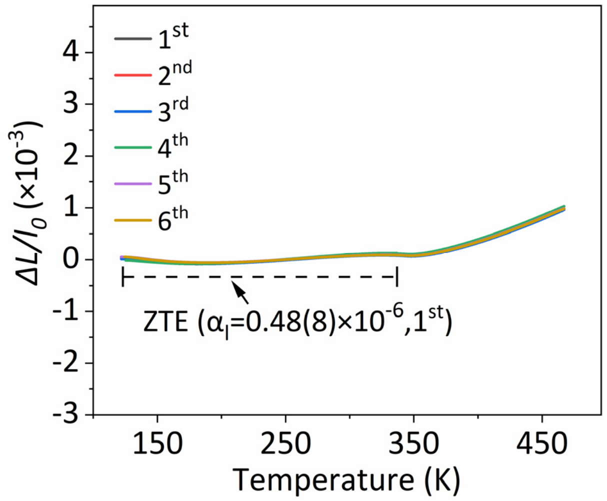

Supplementary Fig. 4. Linear thermal expansion determined by advanced thermo-dilatometer for S-4 along with LD, 1st to 6th refer to six times.

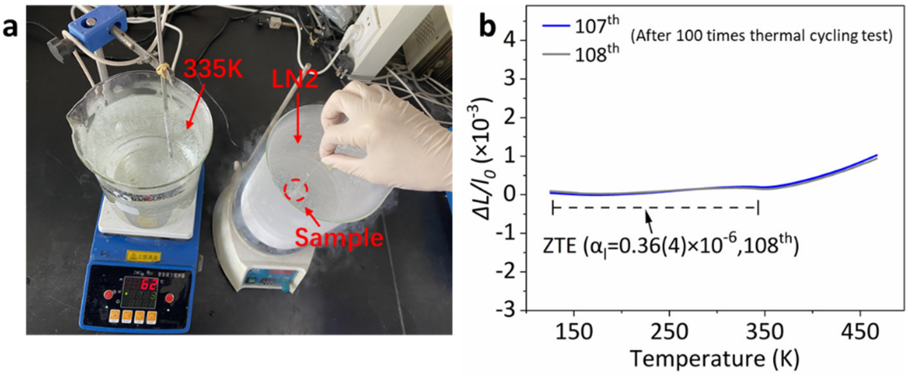

Supplementary Fig. 5. Thermal cycling performance of thermal expansion performance for S-4. **a** Schematic diagram of thermal cycling test. **b** Linear thermal expansion determined by advanced thermo-dilatometer for S-4 along with LD (The 107th and 108th refer to the sample has undergone 100 times thermal cycles).

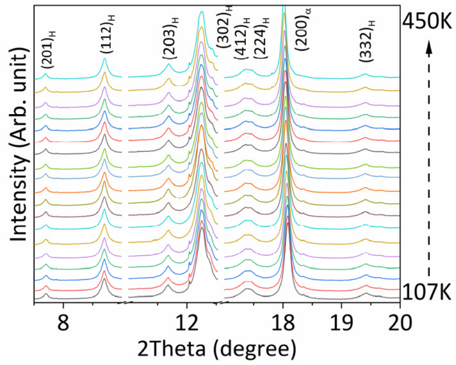

Supplementary Fig. 6. Variable temperature synchrotron X-ray diffraction patterns for S-4 from 107 K to 475 K.

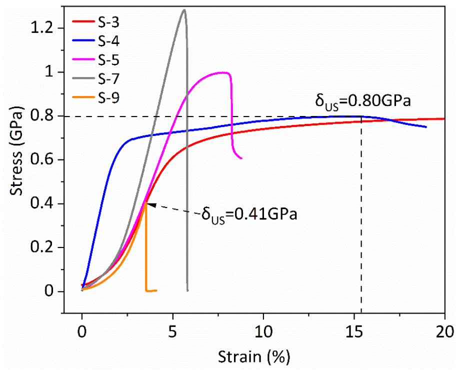

Supplementary Fig. 7. Compressive stress-strain curves for S-3 to S-9.

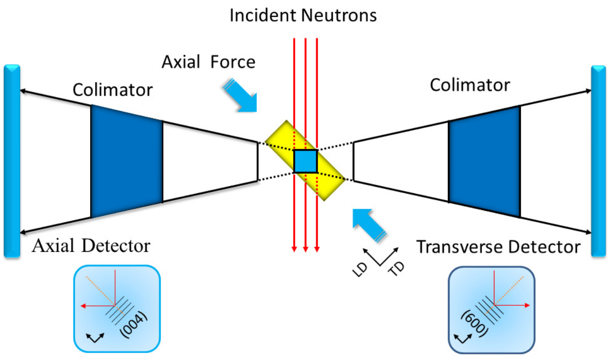

Supplementary Fig. 8. Schematic illustration of the real-time in-situ neutron diffraction experimental set-up on VULCAN from the top of the view. The loading axis is horizontal and positioned at 45° from the incident beam such that Bank 1 probes the strain component along the loading direction (LD), while Bank 2 simultaneously probes the strain component in the transverse direction (TD), as shown in two insets.

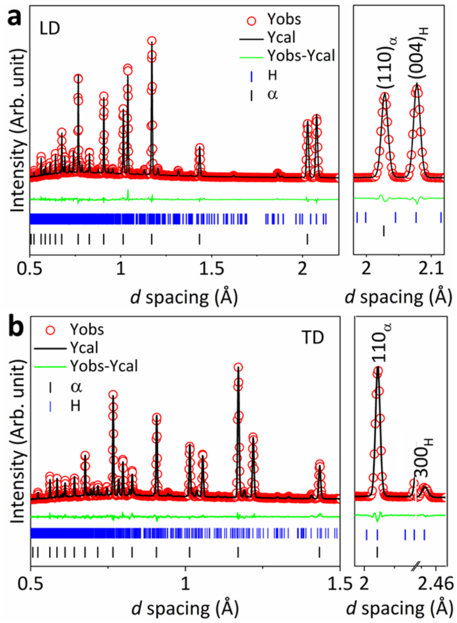

Supplementary Fig. 9. The Rietveld refinements of in situ neutron diffraction profiles of S-4 alloy. **a** Along the loading direction (LD). **b** Along the transverse direction (TD). It shows that $(004)_H$ reflections are significantly strong in LD but almost absent in TD. In contrast, $(600)_H$ reflections are significantly strong in TD, which indicated the S-4 alloy is of high texture along the LD.

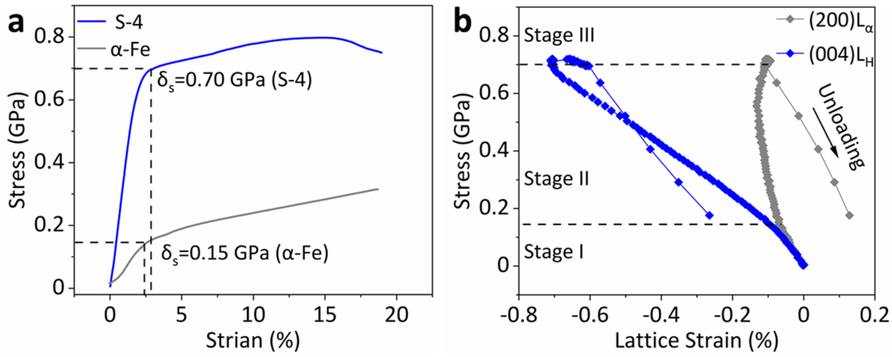

Supplementary Fig. 10. **a** Compressive stress-strain curves of the S-4 and pure α-Fe. **b** Lattice strains in LD versus applied compressive stress for (200)L_α and (004)L_H, respectively.

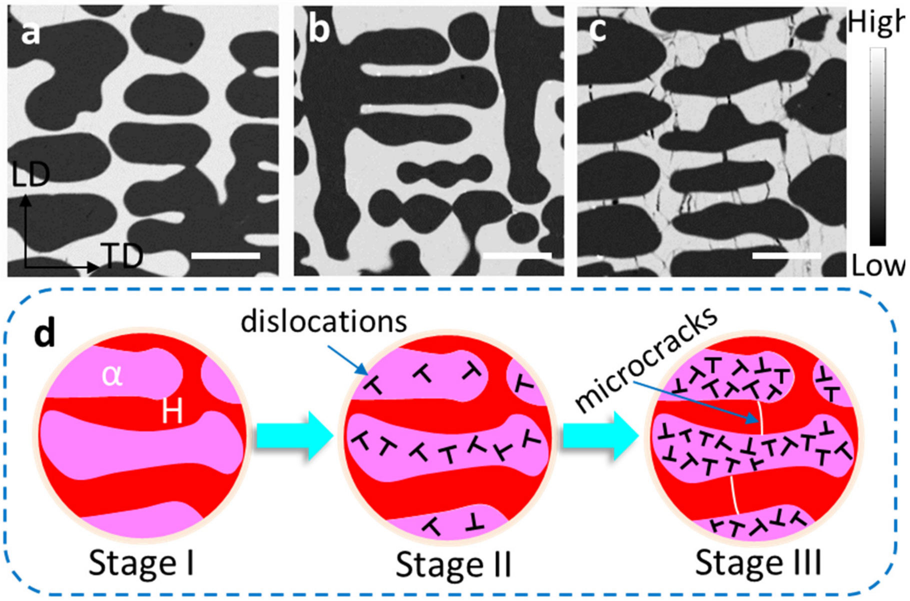

Supplementary Fig. 11. The deformation mechanism of dual-phase alloy S-4. **a**-**c** Ex-situ EPMA along LD at ε= -0% (**a**), -2% (**b**), -12% (**c**), all scale bars represent 25 μm. **d** Schematic illustration of the mechanism of the dual-phase alloys during deformation.

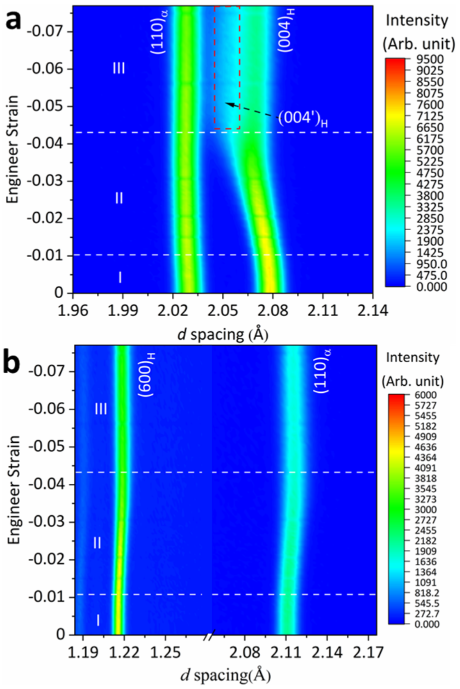

Supplementary Fig. 12. In-situ neutron diffraction profiles of S-4 along (**a**) LD and (**b**)TD.

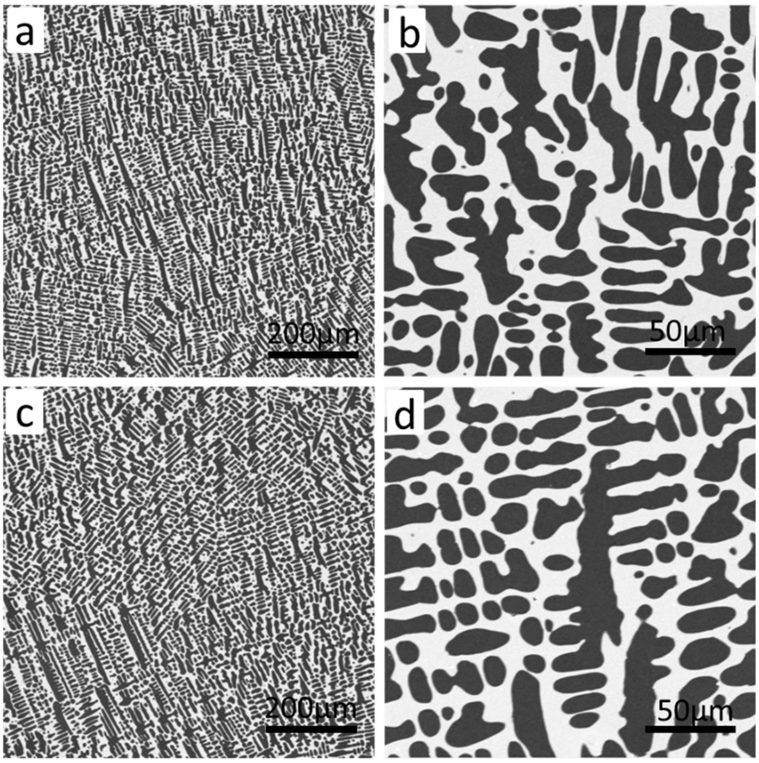

Supplementary Fig. 13. The comparison of microstructures of the as-cast S-4 alloy before and after thermal cycling tests. **a**, **b** The microstructures of the as-cast S-4 alloy before thermal circling tests determined by EPMA in different magnifications. **c**, **d** The microstructures of the

as-cast S-4 alloy after 100 thermal cycling tests (77 K and 335 K) in different magnifications, which shows the alloy maintained perfect integrity and high thermal cycling stability.

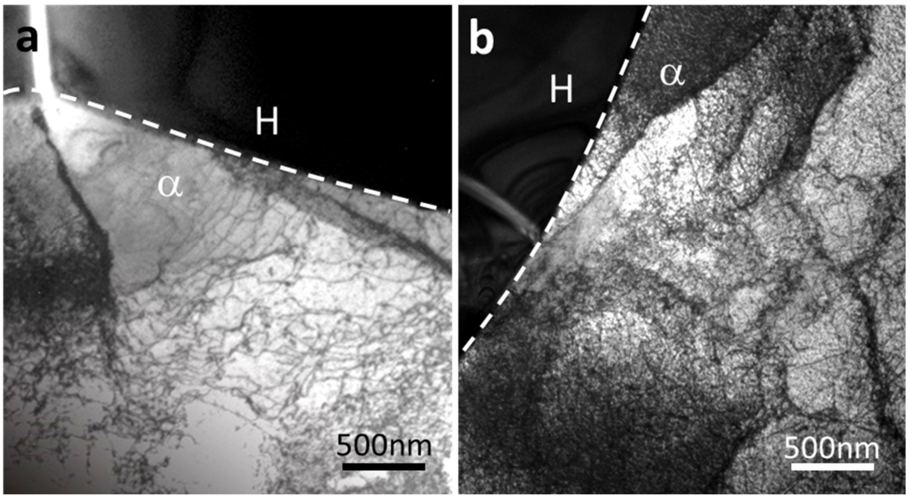

Supplementary Fig. 14. a, b Ex-situ TEM at -12% compressive strain at the dual-phase interface in two separate regions for S-4.

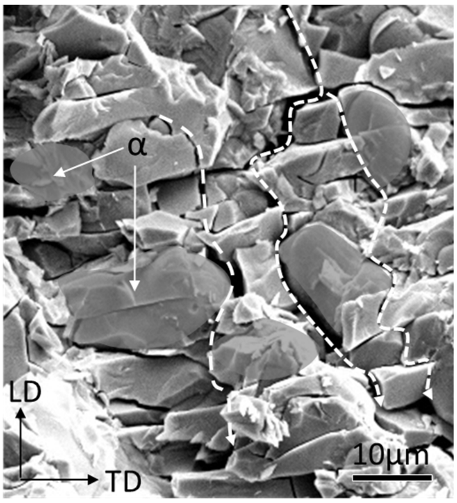

Supplementary Fig. 15. Typical SEM image of the fracture microstructure for S-4, multiple microcracks mainly propagate along with dual-phase interface.

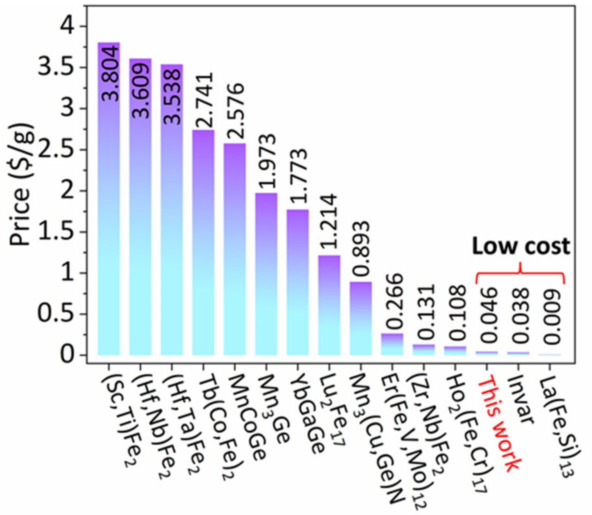

Supplementary Fig. 16. The comparison of typical ZTE functional metal materials. We systematically compared the prices of typical ZTE materials[1-3] (The unit price of raw materials is from Wikipedia 2020 data.). We can see the only widely used invar alloy is much lower than most ZTE / NTE compounds. In the present work, due to the 4% holmium content, the unit price of our ZTE alloy is almost identical in magnitude to Invar alloy. Thus, we defined both our material, $La(Fe,Si)_{13}$ and invar alloy as "low-cost".

Supplementary Table 1. Lattice parameters and structure information of Ho_2Fe_{17} and α - Fe compounds at 300 K determined by SXRD patterns.

Lattice parameter	a	c	V	alpha	beta	gamma
Ho_2Fe_{17}	8.45670	8.31858	515.20	90.0000	90.0000	120.0000
α - Fe	2.93150	2.93150	25.19	90.0000	90.0000	90.0000

Structure	parameter			x	y	z	Occ.	U	Site	Sym.
Ho_2Fe_{17}	1	Ho	Ho2	0.33333	0.66667	0.75000	1.000	0.019	2d	-6m2
	2	Ho	Ho1	0.00000	0.00000	0.25000	0.824	0.019	2b	-6m2
	3	Ho	Ho3	0.33333	0.66667	0.25000	0.112	0.019	2c	-6m2
	4	Fe	Fe1	0.33333	0.66667	0.10676	0.887	0.005	4f	3m.
	5	Fe	Fe2	0.50000	0.00000	0.00000	1.000	0.005	6g	.2/m.
	6	Fe	Fe3	0.33415	0.95614	0.25000	0.757	0.005	12j	m..
	7	Fe	Fe6	0.29823	0.98286	0.25000	0.243	0.005	12j	m..
	8	Fe	Fe4	0.16667	0.33296	0.97899	0.757	0.005	24l	1
	9	Fe	Fe7	0.16667	0.33296	1.00328	0.243	0.005	24l	1
	10	Fe	Fe5	0.00000	0.00000	0.10676	0.177	0.005	4e	3m.
α - Fe	1	Fe	Fe1	0.00000	0.00000	0.00000	1.000		2a	m3m

Supplementary Table 2. Lattice parameters of sample S-4 obtained from SXRD Rietveld refinement.

Temperature (K)	c_H (Å)	a_α (Å)
107	8.3333	2.86279
131	8.33287	2.86324
154	8.33156	2.86377
177	8.33072	2.86442
200	8.32867	2.86502
223	8.32768	2.86567
246	8.32692	2.86624
270	8.32569	2.86696
292	8.32478	2.8675
306	8.32396	2.86807
315	8.32333	2.86836
325	8.32235	2.86864
334	8.32212	2.8689
343	8.32145	2.86925
362	8.32016	2.86983
385	8.31962	2.87057
408	8.32017	2.87138
431	8.3212	2.87222
450	8.32233	2.87288

Supplementary References:

1. Wang, Y. et al. Outstanding comprehensive performance of La(Fe, Si)$_{13}$H$_y$/In composite with durable service life for magnetic refrigeration. *Adv. Electron. Mater.* **4**, 1700636 (2018).
2. Song, Y. et al. Structure, magnetism, and tunable negative thermal expansion in (Hf,Nb)Fe$_2$ alloys. *Chem. Mater.* **29**, 7078-7082 (2017).
3. Song, Y. et al. Transforming thermal expansion from positive to negative: The case of cubic magnetic compounds of (Zr,Nb)Fe$_2$. *J. Phys. Chem. Lett.* **11**, 1954-1961 (2020).
